# Crystallography at CCSU- Twenty years of teaching crystallography to undergraduates at a small regional undergraduate institution.

**DOI:** 10.1063/4.0000801

**Published:** 2025-10-27

**Authors:** Guy Crundwell

**Affiliations:** CCSU, New Britain, CT, USA

## Abstract

Central Connecticut State University is a small public PUI that offers BS degrees in Chemistry & Biochemistry. Twenty years ago, we were successful in obtaining an NSF MRI grant for a single crystal diffractometer. Since then, we have added power diffraction capabilities and published crystallographic data gathered by multiple faculty and myriad students. The XRD has fundamentally redefined our Department, enriched our research, enhanced our collaborations, and, most importantly, allowed undergraduate students access to hands-on crystallographic methods and data refinement. A summary of the in-house and collaborative research of our students is presented.

